# Study protocol: PoPE-Prediction of Preterm delivery by Electrohysterography

**DOI:** 10.1186/1471-2393-14-192

**Published:** 2014-06-05

**Authors:** Hinke de Lau, Chiara Rabotti, Herman P Oosterbaan, Massimo Mischi, Guid S Oei

**Affiliations:** 1 Department of Electrical Engineering, University of Technology Eindhoven, Den Dolech 2, 5612 AZ Eindhoven, the Netherlands; 2 Department of Obstetrics and Gynecology, Jeroen Bosch Hospital, Mailbox 901535200 ME ‘s-Hertogenbosch, the Netherlands; 3 Department of Obstetrics and Gynecology, Máxima Medical Center, Mailbox 77775500 MB Veldhoven, the Netherlands; 4 De Run 4600, 5504 DB Veldhoven, the Netherlands

**Keywords:** Preterm birth, Labor & delivery, Electrohysterogram, Uterine electromyography, Imaging in obs & gyn

## Abstract

**Background:**

Traditional methods used for prediction of preterm delivery are subjective and inaccurate. The Electrohysterogram (EHG) and in particular the estimation of the EHG conduction velocity, is a relatively new promising method for detecting imminent preterm delivery. To date the analysis of the conduction velocity has relied on visual inspection of the signals. As a next step towards the introduction of EHG analysis as a clinical tool, we propose an automated method for EHG conduction velocity estimation for both the speed and direction of single spike propagation.

**Methods/Design:**

The study design will be an observational cohort study. 100 pregnant women, gestational age between 23 + 5 and 34 weeks, admitted for threatening preterm labor or preterm prelabor rupture of membranes, will be included. The length of the cervical canal will be measured by transvaginal ultrasound. The EHG will be recorded using 4 electrodes in a fixed configuration. Contractions will be detected by analysis of the EHG and using an estimation of the intra uterine pressure. In the selected contractions, the delays between channels will be estimated by cross-correlation, and subsequently, the average EHG conduction velocity will be derived. Patients will be classified as labor group and non-labor group based on the time between measurement and delivery. The average conduction velocity and cervical length will be compared between the groups. The main study endpoints will be sensitivity, specificity, and area under the ROC curve for delivery within 1,2,4,7, and 14 days from the measurement.

**Discussion:**

In this study, the diagnostic accuracy of EHG conduction velocity analysis will be evaluated for detecting preterm labor. Visual and automatic detection of contractions will be compared. Planar wave propagation will be assumed for the calculation of the CV vector.

**Trial registration:**

Current Controlled Trials ISRCTN07603227.

## Background

### Traditional methods for detecting preterm labor

Preterm labor and subsequent preterm birth occur in about 10% of all pregnant patients in developed countries and are the leading cause of neonatal mortality and morbidity [1-3]. Antenatal treatment with tocolytics and corticosteroids can be used to improve the neonatal outcome of preterm birth [4]. However, traditional methods used for predicting preterm delivery are subjective and cannot accurately predict when labor will occur. Intrauterine pressure measurement is invasive and cannot be used for pregnancy monitoring. External tocography, being noninvasive, is extensively used for pregnancy monitoring, but provides only quantification of the number of contractions per time unit [5]. Transvaginal ultrasonic cervical length (TVU CL) measurement is widely used in patients presenting with preterm contractions, but it has only a moderate sensitivity and specificity, especially for intermediate values for cervical length [6]. A relatively new biochemical marker, fetal fibronectin, is currently being introduced for short term prediction of preterm delivery. Its strength lies in ruling out imminent preterm delivery, but it cannot be used to predict when labor will occur [7]. Cervical dilatation is a late sign of imminent delivery and the change of successfully delaying delivery using tocolytics is inversely related to the cervical dilatation [8]. Overall more than 50% of the patients admitted for threatening preterm labor, deliver at term [9-11].

### The electrohysterogram and preterm labor

Labor and delivery are preceded by two physiological phenomena: increased excitability and increased connectivity among the myometrial cells resulting in increased propagation of the action potentials that underlie uterine contractions [12-14]. These changes are reflected in the electrohysterogram (EHG), which is a noninvasive abdominal measurement of the uterine electrical activity. As a relatively new diagnostic tool, the EHG has been shown to have potential for monitoring contractions during labor [15-19], as well as detecting pathological contractions leading to preterm delivery [5,20-28]. Various ways of characterizing the EHG have been proposed, including the spectral content using either the peak frequency [5,22-24] or median frequency [28,29] of the power density spectrum, or the ratio between a high and low frequency band [30]. Alternatively, the non-linear correlation among signals in multichannel EHG recordings has been proposed to predict (preterm) delivery [31,32]. The propagation speed of the electrical activity, referred to as conduction velocity (CV), has been quantified by analyzing either the propagation of whole bursts of uterine electrical activity [33,34], or single spikes within a burst [5,21,35,36] Different physiological phenomena could possibly underlie changes in these types of propagation [37,38]. Recently, the estimated CV based on single spike propagation, has been suggested to be a discriminative parameter of imminent preterm delivery [5].

### Towards automated analysis of the CV

To date, analysis of the CV in preterm patients has relied on visual selection of uterine contractile bursts and spikes within these bursts. Despite the promising results that have been presented using this approach, it would be desirable to rely on an automatic approach in order to make the method reproducible and suitable for clinical use. However, automated estimation of the CV entails a number of challenges, namely, automatic detection of uterine contractile bursts, exclusion of signals that are not related to propagating action potentials, and finally calculation of the amplitude and direction of the CV vector. In previous work, we showed automatic calculation of the CV vector within the selected contractile bursts to be feasible [21]. As a next step towards fully automated analysis of the CV in the EHG, we propose an automated method for selecting contractile bursts based on the estimated intrauterine pressure (eIUP) which is derived from the EHG signal [18,21]. Since invasive methods are not available in preterm patients, visual review of the signals will be adopted to refine the automatic burst selection. Furthermore, in order to reduce the complexity and standardize the measurement for use as a clinical tool, a patch with a fixed configuration of five electrodes will be used. This configuration will allow estimating both the speed and direction of the CV amplitude.

In this study protocol we propose an observational study evaluating EHG CV analysis as clinical tool for diagnosing imminent preterm labor using an automated analysis.

## Methods and design

### Study population

The population will consist of patients admitted to the obstetrical ward of the Máxima Medical Center Veldhoven and the Jeroen Bosch hospital for threatening preterm labor or preterm prelabor rupture of membranes and who are eligible for treatment with tocolytics and corticosteroids. The decision on treatment will be based on the standard diagnostic tests and local protocol. Administration of tocolytics will be registered: type and dosage, time of administration.

### Ethics

The study has been approved by the research ethics committee of the Máxima Medical Center and is registered under ISRCTN07603227 in the current controlled trial register.

#### Inclusion criteria

• Gestational age between 23 + 5 and 34 + 0 weeks.

• Clinically evaluated symptoms of preterm labor: at least 6 contractions in 60 minutes based on the external tocogram and/or maternal perception.

• Both singleton and multiple gestations will be included.

#### Exclusion criteria

• Patients in active labor: cervical dilatation >3 cm.

• Signs of infection: baseline fetal heart rate >160 and/or maternal temperature ≥38,0.

• Signs of fetal distress: the following cardiotocogram (CTG) characteristics [39].

○ Baseline heart frequency <100 or >170

○ Reduced variability: <5 bpm during >60 min

○ Complicated variable decelerations, duration >60 sec

Repeated late uniform decelerations.

### Inclusion and measurement

**Figure 1:**
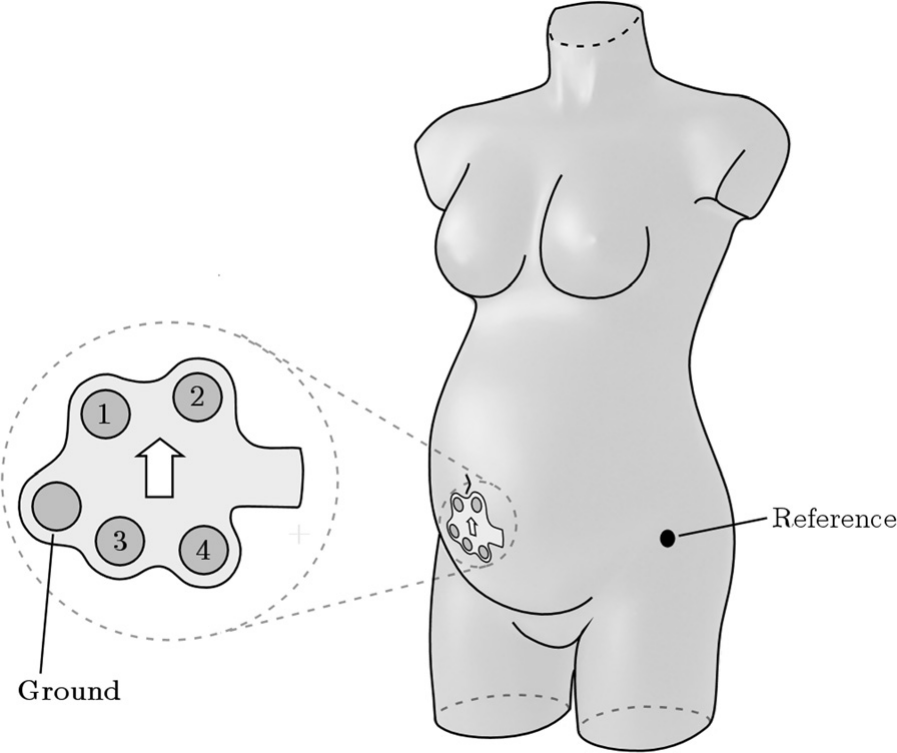
**Schematic drawing of the patch containing four electrodes in a diamond shaped pattern plus a ground electrode.** The patch is placed just underneath the umbilicus in the midline of the maternal abdomen. A reference electrode is placed on the left anterior superior iliac spine.

After written informed consent is acquired, patients will be enrolled in the study. In accordance with local protocol, a CTG registration of at least 30 minutes will be performed and maternal temperature will be measured for all patients. In case of intact membranes a TVU CL measurement will be performed as standard diagnostic test. The length of the cervical canal will be measured in a straight line, the shortest of three measurements will be recorded. Simultaneous with the CTG, the EHG will be recorded using a patch containing 4 monopolar electrodes in a diamond shaped pattern and a ground electrode (Nemo Healthcare B.V.). This patch is placed on the middle of the abdomen, just below the umbilicus. A reference electrode is placed on the left anterior superior iliac spine, see Figure 1. Minimal recording length is 30 minutes. The signals will be amplified and stored on the Porti amplifier (Twente Medical Systems International B.V.). The Porti amplifier will be used in a configuration that does not provide any visible reading of the measurement and the data will be stored directly on its flash memory. All diagnostic tests will be performed within 24 hours after admission.

#### EHG signal analysis

**Figure 2:**
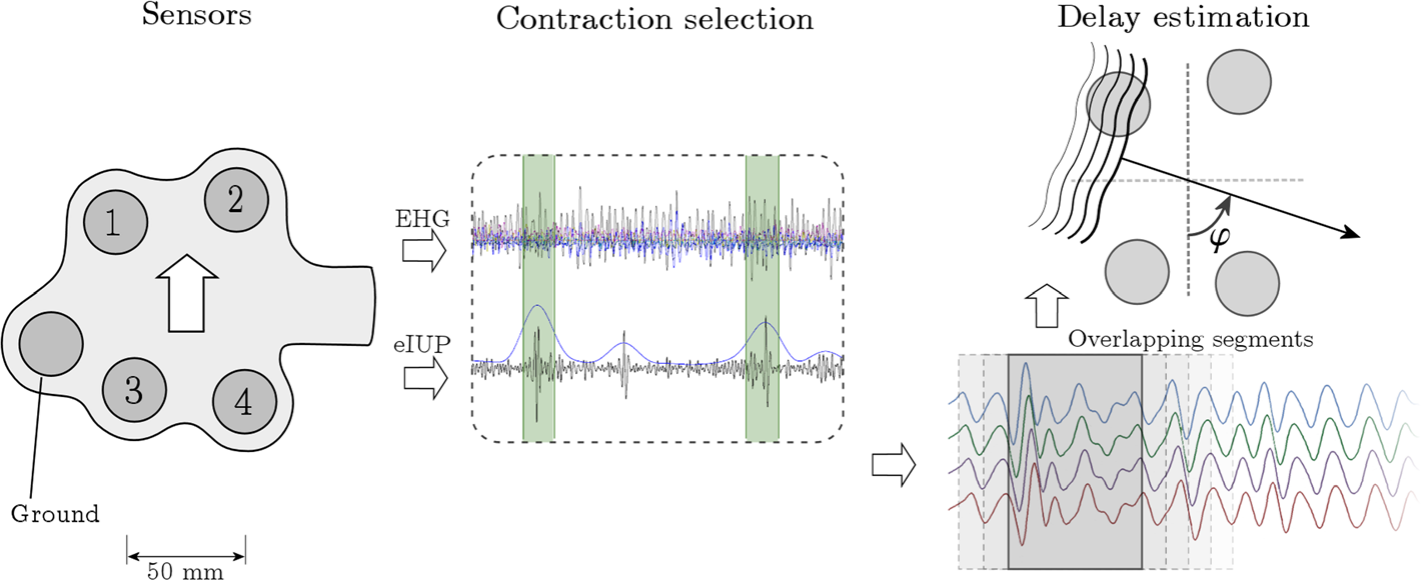
**The method for analyzing the EHG CV.** The EHG is recorded using a fixed configuration of four electrodes plus ground and reference electrodes. The eIUP is derived from the EHG signal and is used for selecting contraction segments. Finally, the delays are estimated in overlapping windows using cross-correlation.

All signal processing and analysis will be performed afterwards offline without knowledge of the pregnancy outcome. Identifying the CV vector will consist of three steps: selection of the contraction segments, estimating the time delays between the channels and calculating the CV vector, see Figure 2. The automatic selection of contractions will be based on the estimate of the internal uterine pressure (eIUP) which is derived from the EHG signal [18]. The algorithm used for the eIUP, as well as the algorithm used for detection of onset and duration of contractions, will be adapted to be suitable for the unpredictable nature of premature and non-labor contractions. Prior to the CV analysis, visual review of the signals will be used to optimize the automatic burst selection.

In the signal segments selected as contractions, the CV will be determined in overlapping windows. The length of these windows will be fixed to a value to allow single spikes to be included. Cross-correlation will be used for estimation of the time delay between channels. We will describe the EHG propagation by a CV vector. This CV vector has an amplitude and an incidence angle with respect to the vertical axis. Based on statistical analysis, a threshold value will be determined for the CV vector amplitude. Values above this threshold will be considered to be artefacts and discarded.

### Study parameters/Endpoints

Patients will be classified as labor group and non-labor group based on the interval between measurement and delivery. The cutoff points will be delivery within 1, 2, 7, and 14 days of the measurement. As primary outcome the EHG parameters will be compared between these groups in terms of amplitude and angle of the CV. In order to be independent of the number of analyzed segments and contractions per patient, the average CV vector will be identified for each analyzed contraction and subsequently the average CV vector for each patient will be determined. As secondary analysis the TVU CL will be compared between the labor and non-labor group. The sensitivity and specificity will be calculated for the EHG parameters and TVU CL for predicting preterm delivery within 1, 2, 7, and 14 days. In order to be independent on arbitrarily chosen cutoff points, the area under the curve of the receiver operating characteristics will be determined.

### Sample size

During the year 2010, a total of 150 patients were admitted to the obstetrical ward of the Máxima Medical Center for threatening preterm labor which met the inclusion criteria. Approximately 40% of these patients delivered within 7 days. A sample size can be calculated based on an observed difference in test characteristics between the standard diagnostics and the EHG analysis using a fixed allocation ratio of 40 – 60% for the positive and negative group respectively. In the study by Lucovnik et al. an area under the curve of the receiver operating characteristics (ROC) of 0,96 was observed for the EHG analysis and 0,72 for the Bishop score (the best performing test among the standard diagnostics) [5].

A sample of 7 from the positive group and 11 from the negative group achieve 82% power, assuming an area under the ROC curve of 0,95 for EHG CV analysis and 0,70 for the standard diagnostics and using a two-sided *z*-test at a significance level of 0.05. This means a minimal number of 20 patients will need to be included. However, this observed difference is based on a single study. In order to increase the likelihood of detecting a difference in case of a lower observed difference or different allocation ratio, we propose to include a total of 100 patients.

### Statistical analysis

Baseline characteristics will be determined for the labor and non-labor group. Differences will be tested for statistical significance using a fisher exact test for dichotomized variables and a non-paired *t*-test for continuous variables. We will evaluate the influence of significant differences in background variables on the outcome using a multivariate logistical regression model.

The Shapiro–Wilk test will be applied to test for a normal distribution of the estimated values of the CV vector amplitude. Levene’s test will be applied to test for equal variances in the labor and non-labor group. An independent samples t-test will be used to test for a significant difference in amplitude of the CV between both groups. The alpha will be set to 0.05 for all statistical tests. Using contingency tables, the sensitivity and specificity of the EHG and standard diagnostics will be determined.

## Discussion

The study aims to evaluate CV analysis of the EHG as clinical tool for the diagnosis of imminent preterm delivery. In an observational cohort study the EHG will be recorded in patients admitted for threatened preterm labor. The CV will be estimated in the EHG using a fully automated analysis. This automated approach entails a novel automatic selection of contractions based on the eIUP and automatic delay estimation. The diagnostic accuracy will be expressed in terms of sensitivity, specificity and area under the ROC curve for delivery within 1,2,7, and 14 days from the measurement.

Similar to [5], part of the patients will already be treated with tocolytics at the time of the measurement. This could influence the amount of contractions and possibly the CV as well. Using logistic regression, the administration of tocolytics will be tested as possible confounding factor. The EHG CV analysis will be evaluated as diagnostic tool for predicting imminent spontaneous preterm delivery. Although it is not a common scenario in patients admitted for threatened preterm labor, patients within the labor group in which labor is induced will not be included in the final analysis.

Perhaps the biggest challenge and also an essential step in accurate estimation of the CV is recognizing contractions amongst noise and artifacts in the EHG. Different from term patients in labor, preterm patients admitted for threatened preterm labor have mostly an irregular pattern of uterine contractions of a varying frequency as well as duration. Therefore, the detection algorithm cannot assume a regular pattern and fixed duration. Furthermore an external tocodynamometer can fail to show contractions in some patients [40,41]. Therefore, for use as independent clinical tool, the EHG CV analysis cannot rely on the external tocodynamometer as reference. Hence the analysis will be mainly based on the electrical signal recorded on the skin.

Our methods will entail filtering in a narrow frequency band (0,3 – 0,8 Hz) in order to suppress signals other than uterine activity, including (abdominal) striated muscle activity and the maternal electrocardiogram. The eIUP will be used in order to distinguish uterine activity from the background noise. Visual review of the data is envisaged to evaluate the performance of the automatic algorithm.

Unlike in our previous work, the time delays between channels will be estimated using cross-correlation. The maximum likelihood method previously proposed, will not be used for this study since it is more suitable when more electrodes are used. Furthermore, given the need for a short time window, no frequency based method can be applied on account of loss of resolution. In addition, it would also increase the complexity and therefore computing time of the algorithm, impeding the use as clinical tool. Using cross-correlation, the resolution in time is dictated by the sampling frequency of the recording and therefore we will use a relatively high sampling frequency of 1000Hz.

The four electrodes of the patch enable the CV vector to be estimated in all the possible directions along the abdominal plane. By using different triangles of electrodes depending on the direction of propagation, the problem of action potentials originating from within the electrodes is mostly solved. Since the dimensions of the electrodes patch are relatively small compared to the whole uterine surface, planar wave propagation will be assumed for the calculation of the CV vector. To explore uterine propagation patterns in detail, an electrode grid containing more electrodes and of a relatively big size would be needed. However this falls beyond the scope of this project, which has the objective to test a clinical application for distinguishing low from high CVs in order to timely recognize imminent preterm labor.

To summarize, in this study the diagnostic accuracy of EHG CV analysis will be evaluated for detecting preterm labor. By employing a fully automated analysis of the EHG without the use of external reference signals, this project aims to make the next step towards introducing the EHG CV analysis as clinical tool.

## Abbreviations

CTG: Cardio TocoGram; CV: Conduction velocity; EHG: ElectroHysteroGram; eIUP: Estimated IntraUterine pressure; ROC: Receiver operating characteristics; TVU CL: Transvaginal ultrasonic cervical length.

## Competing interests

The authors declare that they have no competing interests.

## Authors’ contributions

HdL: study design, ethics approval, measurements, main author of the manuscript. CR participated in the study design, revised the manuscript, setup technical analysis. HPO: principal investigator Jeroen Bosch hospital and responsible for the measurements, revised the manuscript. MM: study design, revised article, supervisor of the technical analysis GO: research design, revised article, supervisor of the project. All authors read and approved the final manuscript.

## Pre-publication history

The pre-publication history for this paper can be accessed here:

http://www.biomedcentral.com/1471-2393/14/192/prepub
